# *Dickeya dadantii*, a Plant Pathogenic Bacterium Producing Cyt-Like Entomotoxins, Causes Septicemia in the Pea Aphid *Acyrthosiphon pisum*

**DOI:** 10.1371/journal.pone.0030702

**Published:** 2012-01-24

**Authors:** Denis Costechareyre, Séverine Balmand, Guy Condemine, Yvan Rahbé

**Affiliations:** 1 INRA, UMR203 BF2I, Biologie Fonctionnelle Insecte et Interactions, INSA-Lyon, Villeurbanne, France; 2 Université de Lyon, Lyon, France; 3 Université Claude Bernard Lyon 1, UCBL Dept Biologie, Villeurbanne, France; 4 INSA-Lyon, Biosciences, Villeurbanne, France; 5 CNRS, UMR5240 MAP, Microbiologie Adaptation et Pathogénie, Villeurbanne, France; University of Wisconsin-Milwaukee, United States of America

## Abstract

*Dickeya dadantii* (syn. *Erwinia chrysanthemi*) is a plant pathogenic bacteria that harbours a cluster of four horizontally-transferred, insect-specific toxin genes. It was recently shown to be capable of causing an acute infection in the pea aphid *Acyrthosiphon pisum* (Insecta: Hemiptera). The infection route of the pathogen, and the role and *in vivo* expression pattern of these toxins, remain unknown. Using bacterial numeration and immunolocalization, we investigated the kinetics and the pattern of infection of this phytopathogenic bacterium within its insect host. We compared infection by the wild-type strain and by the Cyt toxin-deficient mutant. *D. dadantii* was found to form dense clusters in many luminal parts of the aphid intestinal tract, including the stomach, from which it invaded internal tissues as early as day 1 post-infection. Septicemia occurred soon after, with the fat body being the main infected tissue, together with numerous early infections of the embryonic chains showing embryonic gut and fat body as the target organs. Generalized septicemia led to insect death when the bacterial load reached about 10^8^ cfu. Some individual aphids regularly escaped infection, indicating an effective partial immune response to this bacteria. Cyt-defective mutants killed insects more slowly but were capable of localisation in any type of tissue. Cyt toxin expression appeared to be restricted to the digestive tract where it probably assisted in crossing over the first cell barrier and, thus, accelerating bacterial diffusion into the aphid haemocel. Finally, the presence of bacteria on the surface of leaves hosting infected aphids indicated that the insects could be vectors of the bacteria.

## Introduction

Aphids are known vectors of many plant viruses, a feature shared with some of their phloem-feeding relatives, such as whiteflies and scale insects. They are, however, less well known as hosts for pathogenic bacterial infections, although recent surveys have concluded that they universally harbor both obligate and non-obligate bacterial symbionts, referred to as secondary due to their variable prevalence in host species populations. The peculiarity of aphid relationships with their bacterial partners is that these associations are directed by symbiotic mutualistic interactions. Like other vascular-feeding insects, their nutritional ecology is typically dominated by a usually germ-free food, a not-so-common feature for non-parasitic insects. As a result of this situation, the aphid immune system has been shown to lack many homologous components of the pathways described in other insect genomes [1]. The way in which aphids deal with bacterial pathogens, therefore, has been totally overlooked until now, and it is only just starting to be analysed [2].

Recent reports have shown that several plant pathogenic bacteria (*Pseudomonas syringae*, *Pantoea stewartii*, *Erwinia aphidicola* and *Dickeya dadantii*) can also behave as pathogens when they are ingested by the pea aphid. Interactions of these bacteria with the insect have been characterized. *P. syringae* multiplies up to a level of 3×10^6^ colony forming units (cfu) per aphid and the insect succumbs within 48 h [3]. The product of the *fliL* gene, involved in swarming motility, is necessary for full virulence but the reason why swarming is required has not been established. Toxin complex (*tc*) genes, present in the genome, seem to play a limited role in virulence. The excretion of bacteria-containing honeydew shows that the insect may not only be a host but also a vector of the bacteria [3]. *P. stewartii* accumulates in the gut of *A. pisum*, reaching titres of 5×10^8^ cfu, and aphids die within 72 h. The bacteria form aggregates in the hindgut which block the flow of honeydew. A mutant in the *ucp*1 gene, encoding a membrane protein, has a reduced virulence and fails to form aggregates [4]. Although their existence has been established for longer, the interactions between *E. aphidicola* and *A. pisum* are less well characterized. The bacteria seem to multiply in the gut and even though they may be resident without any pathogenic effects, as was initially sampled from established aphid colonies [5], these bacteria have been shown to induce acute pathogenicity in a comparative screening of phytopathogens virulent against the pea aphid [6]. It has been suggested that the production of exopolysaccharides plays a role in this pathogenicity [7].

Artificial infection of *A. pisum* by *D. dadantii,* via an oral route, provokes the death of the insect in about four days. A search, within the bacterial genome sequence, for possible insect toxin genes revealed the presence of a cluster of four genes encoding pore-forming cytolytic toxins, homologous to the Cyt toxins from *Bacillus thuringiensis*, which have probably been horizontally transferred from a GC-poor Gram-positive bacterium [8]. The toxins seem to play a role only when infection occurs by ingestion since a mutant deleted of the toxin genes was as virulent as the wild type strain when infection was performed by injection, a feature frequent in virulence mutants of pathogenic bacteria. This led to the idea that other virulence factors are produced by the bacteria. Several specific and global regulatory mechanisms seem to be involved in the control of the expression of these factors [9]. However, nothing is known as regards how and where the bacteria multiply within the insects. Therefore, we designed the experiments presented here in order to trace infection by *D. dadantii* in orally contaminated pea aphids, with the following main objectives: i) to describe the timing and spatial development of this pathosystem; ii) to identify whether the bacteria are able to successfully infect aphid tissues beyond the initial containment within the intestinal lumen, and to detect whether some tissues are more prone to infection than others; iii) to characterize the pattern of infection of Cyt-defective mutants, in terms of tissue distribution or capacity to cross the first intestinal barrier, when compared to its wild-type counterpart and, finally, iv) to detect the tissue pattern of expression of the Cyt toxins of *D. dadantii*. The methodology used for these characterizations was mainly histochemical, relying on the specific detection of the bacteria through a constitutive surface antigen, the KdgM porin of *D. dadantii*, and the detection of Cyt toxin operon expression [9], with an antibody raised against a purified CytC protein.

## Results

### Bacterial development of *D. dadantii* in aphids

Our previous study [6] showed that when aphids were fed, for 24 h, on AP3 medium containing 10^6^ bacteria/ml and then replaced on plants, the survival curve varied according to whether the *D. dadantii* wild type strain (A3952) or the mutant deleted of the four cyt-like toxin genes (A4977) was used. With the wt strain, aphids began to die after 24 hours and, after 4 days, only 30% had survived. With the *Δcyt* strain, aphids started to die after 3 days and 40% had survived at the end of the experiment.

To determine whether the survivors had eliminated bacteria or whether they did not, in fact, ingest any, the number of bacteria was counted in aphids fed with AP3, containing 10^6^ bacteria/ml of strain A3952, for 24 hours (day 1). All aphids (n = 20) contained bacteria, but their number showed great variability, ranging from 300 to 2×10^5^ cfu. This confirmed, however, that all aphids had ingested bacteria. Next, the aphids were placed onto broad bean plants and a time-series of bacterial numeration was performed, counting the number of bacteria in aphids every day until no new deaths occurred. 24 hours later (on day 2) we found that about 10% of the live aphids were free from *D. dadantii*. This percentage increased over the following days (about 30% on day 3 and about 60% on day 4) to reach 100% of the surviving aphids on day 5. By this time, all the aphids that could not eliminate bacteria were dead. In summary, 30% of the initial number of aphids were able to eliminate the infecting *D. dadantii*.

**Figure 1:**
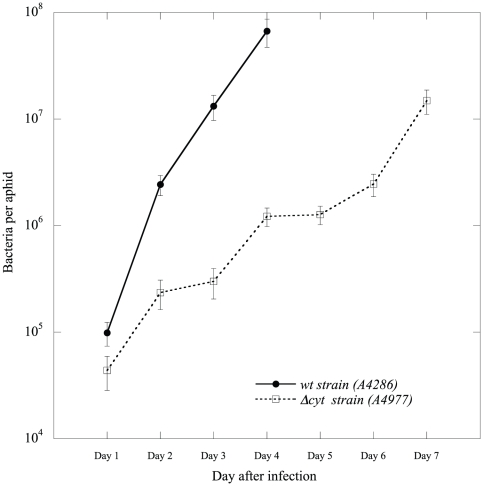
Bacterial population growth in aphids. Numeration of *D. dadantii* populations within infected pea aphids. *wt*: infection with wild-type *D. dadantii*; *Δcyt*: infection with strain A4977, with the four *cyt* genes deleted. Data are means ± SE of bacterial numerations (n from 6 to 28, depending on day and survival rates). Ingestion dose was 10^6^ cell/mL. Only aphids still infected with bacteria were included in the counts.

In infected aphids, the average number of bacteria was generally 10-fold higher on day 2 than on day 1, but the variability was still significant (Fig. 1). After day 2, the number of bacteria continued to increase regularly but more slowly, and it reached a maximum value of about 10^8^ bacteria/aphid on day 4 (Fig. 1).

The same experiments were performed for aphids infected with the *Δcyt* strain. The number of bacteria in aphids fed with AP3 inoculated with A4977 strain, for 24 hours, was monitored as previously. All aphids (n = 28) on day 1 contained bacteria but, again, their number showed a high variability, ranging from 240 to 3.9×10^5^ cfu. This test confirmed, once again, that all the aphids had ingested bacteria. As for the wt strain, the average number of bacteria in infected aphids was 10-fold higher on day 2 than on day 1, but variability was still significant. After day 2, the number of bacteria increased continuously, though much slower than for the wild-type series, reaching a maximum value of 3×10^7^ bacteria per aphid (Figure 1). No new deaths were observed after day 7, and the surviving aphids (about 40%) were always found to be devoid of bacteria and were considered as having escaped infection.

Finally, in none of the haemolymph samples collected at all stages of infection were we able to detect viable *D. dadantii* cells, probably indicating that the transfer of bacteria through haemolymph was a very transient state and, probably, no stable multiplication of *D. dadantii* cells occurred in the blood cells.

### Histological localization of *D. dadantii* in infected aphids

In order to determine the localization of *D. dadantii* after oral ingestion of the bacteria, immunofluorescence detection of whole aphid mounts was carried out. In general, transverse sections of infected aphids were cut and then stained using an antibody raised against a *D. dadantii* outer membrane protein, the oligogalacturonate-specific porin KdgM. KdgM staining is very specific to this species, and no positive staining could be observed in the control non-infected insects (Fig. S1). We observed that bacteria were present in many somatic tissues, and that they could reach the reproductive system and potentially invade parthenogenetic embryos quite early on in the infection process.

**Figure 2:**
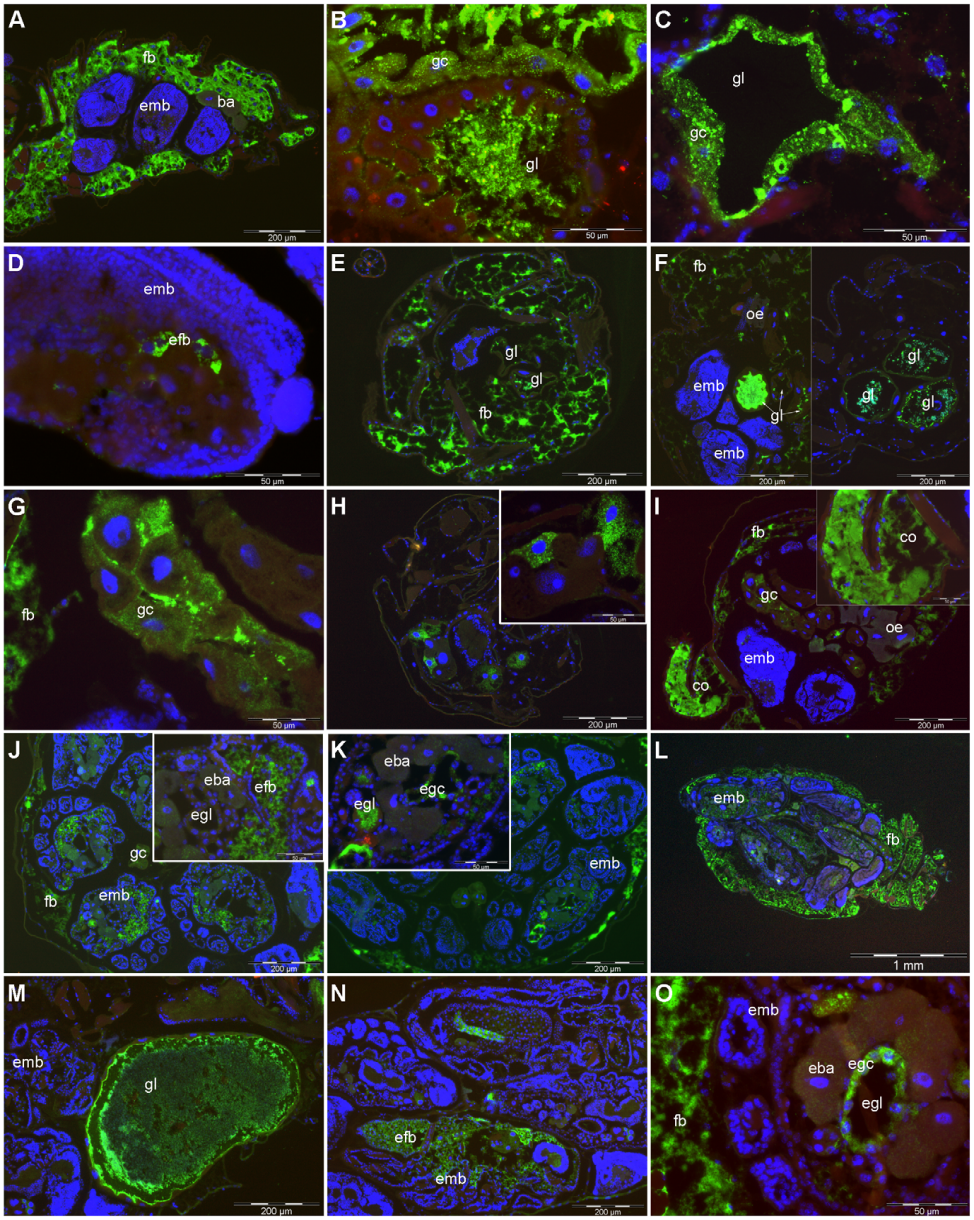
Pattern of aphid tissues infected with *D. dadantii*. Immunostaining with anti KdgM antibodies of aphids infected with the wild type *D. dadantii* strain. Anti KdgM antibodies were detected with anti IgG labeled with Alexa fluor 488 (green labeling) and DNA was stained with DAPI (blue labeling). **2A to 2D**: early infection stage: day 1 of infection (aphids collected on infected diet). *D. dadantii* is located mainly in the fat body (fb, 2A), within the gut lumen (gl, 2B), and can also be detected in some gut cells (gc, 2C) as well as in the embryonic fat body (efb, 2D). **2E to 1K**: early infection stage: day 2 post infection. *D. dadantii* is located as a general infection of the fat body (2E), as dense aggregates in the gut lumen (2F), in gut cells (2G) and, occasionnally, in all of the following tissues: brain (2H), cornicles (co, 2I) and in many embryos showing either embryonic fat body (efb, 1J) or embryonic gut infections (egc egl, 2K). **2L to 2O**: late infection stage: day 4 post-infection. A heavy infection of *D. dadantii* is seen in all the maternal and embryonic fat bodies (2L), the gut tissue and lumen (2M), and large parts of the embryonic fat body (2N) and embryo gut cells, but not in the embryonic bacteriocytes (egc, eba, 2O). Abbreviations - ba: bacteriocytes, brc: brain cells, co: cornicles, emb: embryo, eba: embryonic bacteriocyte; efb: embryonic fat body, egc: embryonic gut cells, egl: embryonic gut lumen, fb: fat body, gc: gut cells, gl: gut lumen, oe:eonocytes.

During the very early stage of infection, when insects were still feeding on inoculated diets (day 1), bacteria infected the aphid by entering and multiplying within the gut lumen. At this point, they may infect gut cells and be detected within the cytoplasm of some cells, though this was not often observed at this stage (Fig. 2A, B, C). They were also spreading throughout the fat body in a limited number of cases (Fig. 2A). In a few aphids, *D. dadantii* was also detected in the fat body of embryos within the first day of contact with the insect (Fig. 2D).

Afterwards, during the primary stage of infection (day 2), bacteria disseminated across various tissues within the aphid body. They were always detected within the lumen of the gut, sometimes in deeply stained spots (Fig. 2E, F). They had also, very frequently, spread throughout the fat body, as shown in a typical transverse section of a whole aphid nymph (Fig. 2E). In some aphids, gut cell cytoplasm showed intracellular fluorescence, signaling the presence of the bacterium within the cell (Fig. 2G), although extracellular gut lumen location was by far the most common position of the infecting bacteria (Fig. 2F, left and right). *D. dadantii* was also occasionally observed in some brain cells (not all, Fig. 2H inlet), as well as in the cornicle of the aphid (Fig. 2I). In those insects, almost all the body tissues were infected, corresponding to an effective septicemia. It should be noted that some tissues contained a much lower signal than the “standard” infected tissues, such as the fat body, including the bacteriocytes and some large isolated cells identified as oenocytes (Fig. 2I). However, at a higher magnification, bacteriocytes showed some cytoplasmic autofluorescence (present in control insects) which prevents us from claiming a complete exclusion of *Dickeya* cells in these bacteriocytes, although the level of staining was always very much reduced compared with the gut and fat body cells (Fig. 2J, K). At this primary stage of infection, many embryos were infected, not only within the fat body but also in the gut cells and, less frequently, in the luminal embryonic gut (Fig. 2J, K,L).

Finally, during the late stage of infection (day 4 post-infection), staining revealed an even larger number of bacteria present in the gut lumen of aphids, with occasionally concomitant gut cell penetration showing parts of the gut, frequently the anterior mesenteron (often termed “stomach”) completely filled with *D. dadantii* (Fig. 2M). Additionally, at this late stage, where mortality had already occurred in more than half of the infected individuals, *D. dadantii* extensively invaded the fat body and the embryos of all infected aphids (Fig. 2L, 2N). Noticeably again, embryonic bacteriocytes from positively stained embryos never showed any positive staining, whilst the neighbouring gut cells and fat body tissue did (eba *vs* egc, Fig. 2O).

### Expression of CytC toxin in *A. pisum*

**Figure 3:**
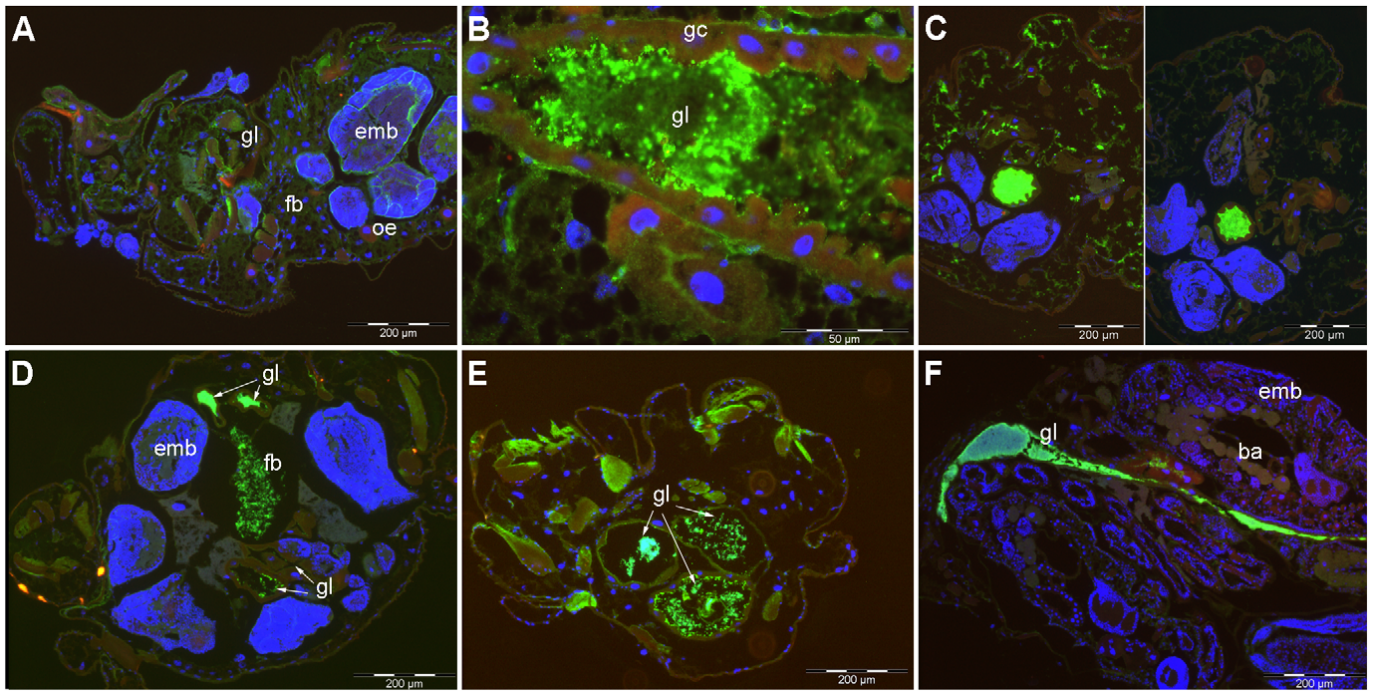
Expression of CytC toxin in aphid tissues. Immunostaining with anti CytC antibodies (except 3C, left) of aphids infected with the wild type *D. dadantii* strain. Anti CytC antibodies were detected with anti IgG labeled with Alexa fluor 488 (green labeling) and DNA was stained with DAPI (blue labeling). **3A to 3B**: early infection stage: day 1 of infection. In most samples, no expression of the toxin was detected (3A) although bacterial presence was readily observed in the same sample, for example in the gut lumen (see Fig. 2); in some individual insects, toxin expression was detected in the gut lumen (3B). **3C to 3E**: Primary infection stage: day 2 post infection; 3C, left and right: typical staining difference (in the same aphid) between bacterial detection at many locations, including the fat body (anti-KdgM antibody, 3C left) and toxin expression detection, restricted to the digestive tract (anti-CytC antibody, 3C right); In some instances, toxin expression was also detected in clustered areas of the fat body, in addition to typical gut lumen localization (gl, fb, 3D–E); **2F**: late infection stage, day 4 post-infection. Toxin detected as a marked stain only in the gut lumen (2F). Abbreviations as in Figure 2, plus: oe: oenocytes.

Expression of CytC has also been investigated by immunofluorescence, using an antibody raised against the toxin. The specificity of antibody was checked (Fig. S2). Overall, when analyzed systematically on the same aphid samples as those used for *D. dadantii* detection of KdgM, visualization of the toxin was much more restricted. Although detected in one sample from the early infection stage (day 1, Fig. 3B), the toxin was more detected in later stages when the bacteria were visible as dense markings in the gut lumen. The most common location, by far, for toxin expression was the gut lumen, even when the bacteria were massively detected elsewhere in the aphid tissues (Fig. 3C). Generally no expression of the toxin was observed in the fat body, although some staining in partial areas of this tissue was occasionally detected (Fig. 3D). Extensive staining outside the gut lumen only appeared exceptionally (Fig. 3E), and even at late infection stages (day 4 to day 7), where bacteria were shown to be widespread in the aphid body (as shown in Fig. 3C left), the typical localization of the toxin stain was as high density signal areas within the gut lumen (Fig. 3F). It is noteworthy that no signal for toxin expression was visible in embryonic tissues, in contrast to the observations of bacterial localization.

### Histological localization of *Δtox D. dadantii* in infected aphids

**Figure 4:**
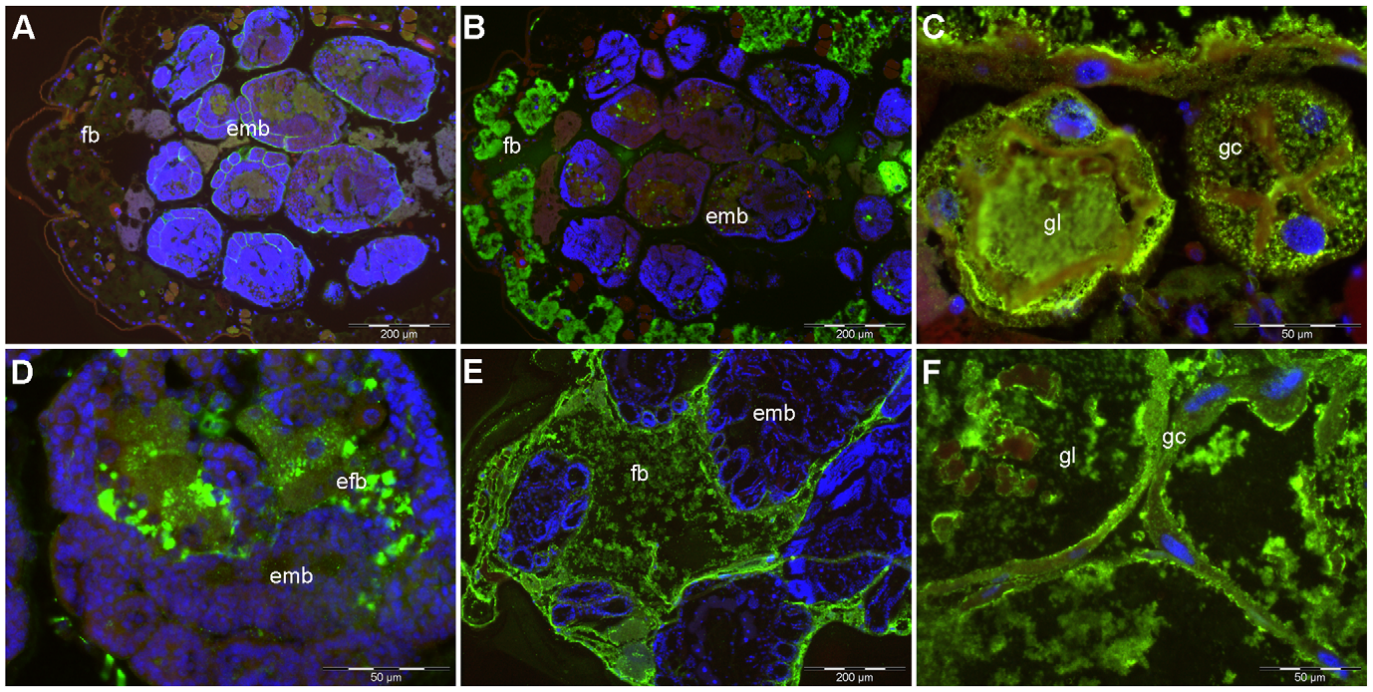
Infection of pea aphids by the toxin defective mutant strain. Immunostaining with anti KdgM or anti CytC antibodies of aphids infected with *D. dadantii* strain A4977. Anti KdgM and anti CytC antibodies were detected with anti IgG labelled with Alexa fluor 488 (green labelling) and DNA was stained with DAPI (blue labelling). **4A**: Staining with anti-CytC antibody of an aphid at late infection stage (day 4 post infection). Stain was never detected on such mutants. All the following images are with anti-KdgM stain to detect *D. dadantii* cell localization. **4B to 4D**: early infection stage (day 1). When detected, bacterial cells were localized at standard sites, as compared to the wild-type strain, such as in the fat body (4B), the gut lumen and gut cells (4C), and even in the embryonic fat body (4D); **4E to 4F**: late infection stage, day 4 post infection. *D. dadantii* are detected in the maternal fat body (4E), and in the gut lumen and cells (4F). Abbreviations are as in Figure 2.

In an attempt to determine whether the absence of Cyt-like toxins impacts qualitatively on the tissue distribution of the infecting bacteria, we also observed the spread of bacteria in aphids after toxin gene deletion. Oral infection was conducted with the mutant *D. dadantii* strain A4977, lacking the cluster of four toxin genes. As expected, no signal was observed using anti-CytC antibody (Fig. 4A), which confirmed the specificity of the detection tool. With anti-KdgM immunostaining, we were able to see that *D. dadantii* dispersal throughout the aphid body did not seem to be qualitatively affected by the absence of the toxins. Indeed, as was observed in wild-type aphids, bacteria were shown to be present in large numbers within the gut lumen (Fig. 4C, 4F), as well as occasionally within gut cells (Fig. 4C). *D. dadantii* cells were also widely seen infecting the fat body (Fig. 4B, 4E). Embryos also seem to be affected by mutant bacteria as we were able to detect infection in the embryonic fat body of an aphid during the early infection stage (Fig. 4D). Although the quantification of differences between modalities was difficult in our histology screening, this study revealed no clear distinction between the tissue distribution of the mutant strain and its wild-type relative.

### Can *A. pisum* be a vector of *D. dadantii?*

To determine if the aphid is not only a host but also a putative vector of *D. dadantii*, we tested whether infected insects can disseminate bacteria. We did not detect any bacteria in newborn aphid nymphs laid from an infected batch (n = 30), *i.e.* from individuals escaping from a lethal infection by *D. dadantii*. We also observed that insects which were clearly infected, as identified by their biological phenotype (reduced mobility and slightly different color), were never able to produce nymphs. No evidence of vertical transmission of the bacteria was, therefore, found in our experiments.

To investigate whether viable bacteria can be detected on the leaf surface of *V. faba* that have hosted infected aphids, leaves from the plants were harvested three days after the removal of all aphids (whether dead or escaping infection). Leaves were rinsed with M63 medium and bacteria were counted on selective medium. Bacterial titers from 1.0×10^3^ to 1.9×10^6^ bacteria per leaf (1.5×10^2^ to 2.4×10^5^ bacteria cm^2^) were found with an average titer of 3.0×10^5^ bacteria/leaf (3.8×10^4^ bacteria/cm^2^).

Finally, we counted the number of viable bacteria present in dead aphid bodies in a daily time series. The survival of bacteria at 20°C, within this micro-ecosystem, could extend to one week, with a progressive decrease in viability: nearly 5.10^5^ bacteria were surviving five days after the death of the aphids (Table 1). Thus, the aphid is not necessarily the end point for the bacteria, and it can be regarded as playing a potential role in the further dissemination of *D. dadantii*.

**Table 1:** Bacterial survival and decay in dead aphids.

Time after death	CFU^a^
Day 0 (death)	7 10^+7^±9 10^+6^
Day 3	3.1 10^+6^±7 10^+5^
Day 5	4.5 10^+5^±2 10^+5^
Day 7	1.3 10^+2^±6 10^+1^
Day 10	0±0

a Bacterial numeration, mean ± SE (n = 4).

## Discussion

*A. pisum* interacts with different types of bacteria. The obligatory symbiont *Buchnera aphidicola*, contained within specialized bacteriocytes, provides the insect with essential amino acids. Facultative symbionts, such as *Hamiltonella defensa*, *Regiella insecticola* or *Serratia symbiotica* can also provide diverse benefits to their host [10]. These bacteria are found either in the bacteriocytes or in the haemolymph. To co-exist with these bacteria, it has been proposed that A. *pisum* has a limited immune system which permits the maintenance of these bacteria but, as a consequence, this could render it more prone to microbial infections [1]. Recently, several bacteria that are lethal to aphids have been described. These bacteria are either plant pathogens, such as *P. stewartii* or *P. syringae*, that may infect feeding insects, or the non-pathogenic model bacteria *Escherichia coli* [3], [4], [11]. However, the mechanism by which they kill the aphids is not yet understood. When the route of infection is ingestion, which is probably the natural way of infection, these bacteria multiply in the insect gut. They do not seem able to cross the gut epithelium and were not found in the insect body cavity. It seems that, at least in the case of *P. stewartii*, death occurs by accumulation of bacterial aggregates in the crop and the gut. Infection by *D. dadantii* seems to follow a different path. Initially the bacteria multiply abundantly in the gut but, by as early as one day post-infection, they have crossed the gut epithelium and can be found in the body cavity of the insect, with a preferential localization in the fat body. As infection progresses, other organs, such as the brain or the embryos, are invaded and it is probable that death is provoked by this septicemia. The means by which the bacteria spread so quickly is still not known since, surprisingly, no living bacteria have been found in the haemolymph. Laughton *et al* [12] have shown that the haemolymph contains granulocytes that phagocytose very efficiently any bacteria and destroy rapidly circulating cells. This cellular immunity may be the main reason for our inability to recover viable bacteria from haemolymph samples, as it is difficult to think of a rapid infection route to the fat-body or to the embryonic chain without any transit time in the general circulation. Due to the very rapid time-course observed, an alternative cell-to-cell route of infection seems unlikely.

Apart from the Cyt proteins, the genome of *D. dadantii* does not encode homologues of other known insect toxins. However, our results suggest that these toxins are not the main factor responsible for insect death. CytC was strongly detected in the gut but was barely visible in the body cavity at stages where it was full of bacteria. Our previous work showed that *cyt* gene expression was induced in a high osmolarity medium [9]. Insects feed on phloem sap, or AP3 medium, that contains a high sucrose concentration. This could induce Cyt production in the gut. Once the gut epithelium has been crossed, bacteria are in lower osmolarity conditions and Cyt synthesis would go back to a basal level, which is no longer detectable by immunodetection. The fact that the bacteria present in the embryo gut do not produce CytC confirms that this expression depends on the feeding medium, and not on localization in a specific organ. *B. thuringiensis* Cyt toxins are viewed and classified, by homology, as haemolytic pore-forming toxins that could insert into the gut epithelial cell membrane and provoke the formation of holes, ultimately leading to cell death. Bacteria could use this pore-forming mechanism to enhance their epithelial penetration ability, as has been shown with Cry toxins from the same *B. thuringiensis*, which clearly exhibited such septicemia-enhancing properties in a specific host-pathogen context [13], [14], [15], contrasting with its canonical cell-toxicity phenotype. It is possible that the role of *D. dadantii* Cyt toxins is the same since a *Δcyt* mutant is as virulent as the wt when infection is induced by injection [6] and we observe only a delay in the death of the insect aphids with the mutant, but with the same pattern of general infection. The precise factor responsible for the insect mortality has yet to be identified. It should be noted, however, that although virulence factors active against the pea aphid have been identified in *D. dadantii* [9], insect death may result from a complex physiopathological syndrome resulting from the impairment of central metabolic functions following infection, as has been discussed for the fruit fly [16]. The generalized infection of the fat body, observed in our model, could reflect such a situation.

*D. dadantii* is classified as a necrotrophic plant pathogen: its principal virulence factors are plant cell wall degrading enzymes, mainly pectinases, that destroy the pecto-cellulosic matrix that holds the cells together, leading to their death by osmotic shock [17]. This gives the bacteria wide access to nutrients. Interactions with animal cells may occur differently. It has been shown that *D. dadantii* is able to adhere to, and kill, human adenocarcinoma cells [18]. The Hrp type III secretion system seems to be involved in this phenomenon, suggesting that this phytopathogenic bacteria has animal-directed toxins. In the case of aphid infection there is no indication of tissue disorganization, which would be a sign of massive lytic enzyme secretion by a type II secretion system. Instead, toxins may be delivered by the type III Hrp secretion system or the type IV secretion system, present in the genome but of unknown function. No secretion signal whatsoever has been detected in the proteins from the *cyt* operon.

Most infection experiments finish with a low, though significant, proportion of individuals that have escaped infection. These aphids have always been shown to be devoid of viable *D. dadantii* cells, and this could reflect the fact that an efficient immune reaction was mounted against the pathogen. Despite the fact that genomic and experimental data have repeatedly shown that aphid immunity is somehow atypical among insects and lacking in many essential components, such as the *imd* pathway or an efficient peptide antibacterial response [1], [11], the aphids that escape infection, together with recent reports on the identification of immunocompetent cells in aphid haemolymph [12], are clear indications that an immune response is active during bacterial infection of the pea aphid. The observation of a differential sensitivity of the tissues (eg. bacteriocytes versus fat body) is another indication of tissue-specific defense factors. An example of this is the highly expressed lysozyme gene that has been shown to dominate the expression pattern of aphid bacteriocytes [19], [20].

The mode of transmission of pectinolytic enterobacteriae (*Pectobacterium* and *Dickeya*) is not clear. The role of soil, water and the agricultural methods used has been demonstrated [21] but a possible involvement of insects has received little attention, although studies have revealed the transmission of *Pectobacterium* to potato plants by *Drosophila* [22]. We present evidence here that aphids can be not only hosts but also vectors of *D. dadantii*. Whilst infection can occur with as little as 100 bacteria, more than 10^6^ bacteria deposited by honeydew may be found on leaves where infected aphids have fed and this could be a new source of contamination. Moreover, dead insects, that may fall on the soil or be disseminated by the wind, contain live bacteria for as long as one week. Thus, the role of insects in *D. dadantii* transmission needs to be re-assessed.

## Methods

### Bacterial strains and medium

The *D. dadantii* strains used in this study are A4977 (*Δcyt ABCD*-KanR) [9] and A4286 (*ganB*-KanR) [23]. This latter strain is a *D. dadantii* 3937 mutant that is used to follow bacterial growth in aphids. This mutation does not modify fitness or bacterial virulence against aphids. Bacteria were grown in LB, M63 or AP3 medium, which is the standard diet used for aphid artificial bioassays [24]. To exclude any effect of a differential growth rate between the two strains, we measured this parameter in AP3 medium and in M63 medium with 0.2% sucrose. The growth rate was equivalent for all the strains. Bacterial dilutions were performed in M63 medium and plated on LB plates containing kanamycin at 25 mg/l.

### Insects and insect bioassays

The aphid clone used was LL01, an alfalfa collected clone long-established for use in the laboratory, and it was grown on *Vicia faba* (cv. Aquadulce). This genotype has been used in all our previous work on *A. pisum*/*D. dadantii* interactions, and the injection, ingestion and survival analyses have been fully described elsewhere [6].

### Numeration of bacteria in infected aphids and in aphid haemolymph

Inoculations were performed as described in Grenier *et al.* [6]. For bacterial numeration of insects inoculated by ingestion, third instar aphid nymphs, fed on broad beans, were maintained for 24 h on an AP3 diet, containing bacteria at 10^6^ bacteria/ml, before being placed back onto bean leaves at 20°C. Aphids collected at this time were recorded as “day 1”. Each day, several survivors were used to perform histological analyses or to follow bacterial multiplication. In the latter case, aphids were crushed in a microcentrifuge tube, with a sterilized pestle, in 1 ml of M63 medium. Serial dilutions were plated on LB+kanamycin plates, and incubated at 28°C. In aphid survivors, the presence of ingested bacteria in the circulatory system was also monitored by sampling haemolymph, using a capillary through a leg cut. For each assay, the haemolymph of ten aphids was pooled prior to dilution in 100 µl of M63 medium, and then plated on selective LB plates.

Additional bacterial detection experiments were performed with aphids infected by a standard oral inoculation with the wt strain, from which haemolymph was collected at all stages of infection, and samples then incubated on selective plates.

### Detection of *D. dadantii* in newborn aphids and on plant material

For the numeration experiment, third star nymphs, fed on broad beans, were maintained for 24 h on an AP3 diet with 10^6^ bacteria/ml (strain A4286) before being placed back onto bean leaves at 20°C. After laying, three batches of ten newborn aphids were collected and then squashed, in a microcentrifuge tube, in 1 ml of M63 medium. For the detection of bacteria on plant material, four leaves per plant were harvested and washed in 10 ml of M63 medium. Leaf surface area was calculated by tracing the leaves on grid paper. Serial dilutions were plated on LB+kanamycin plates added with cycloheximide at 50 µg/mL, and incubated at 28°C to detect the presence of *D. dadantii*. Experiments were performed twice.

### Production of the CytC and KdgM antibodies

*cytC* was amplified with the oligonucleotides CytC+ (5′cctgggatccaacaatattgcattgaatccga 3′) and CytC- (5′ gccgctcgagggttgatagatccagtctgcc 3′). The amplified DNA was digested with BamHI and XhoI and ligated into pGEX-6p3 (GE Healthcare), digested with the same enzymes. The GST-CytC protein was produced in *E. coli* NM522 cells grown in LB medium+1 mM isopropylthiogalactoside. Cells were collected by centrifugation and then broken in a French cell press. Unbroken cells were eliminated by centrifugation. GST-CytC was bound on GST-sepharose and the CytC protein was liberated by the addition of Prescission® protease, according to the manufacturer's protocol. The protein was injected into a rabbit, for antibody production, by Valbex (Villeurbanne, France). The KdgM antibody used in this study was produced following the protocol described in a previous study on this porin [25]. This antibody was used as a marker of *D. dadantii* [8] in the aphid tissues, as the aphid primary symbiont *Buchnera* does not harbor a KdgM homolog and, in addition, our LL01 clone is devoid of any aphid secondary symbiont.

### Sample preparation for histology

Antennae and legs were removed from the aphids prior to fixation of the rest of the body in Finefix solution (Milestone, Bergamo, Italy). After one week at 4°C, fixative was replaced by several baths of PBS before embedding the aphids in agar at 1.3%. Samples were then dehydrated, through a graded ethanol series, and moved into butanol-1 at 4°C for one week. The aphids in agar were then impregnated and embedded with melted Paraplast. Wax blocks were kept dust free until sectioning. Tissue sections, 5 µm thick, were cut using a LKB Historange microtome, then sections were placed on poly-lysine coated slides, dried overnight in a 37°C oven and kept at 4°C until immunostaining.

### Immunostaining and microscopy

Paraffin sections were dewaxed in 2 baths of methylcyclohexan for 10 minutes, rinsed in 100° ethanol, and then rehydrated through an ethanol gradient to PBS. Slides were incubated with 1% BSA in PBS for 30 minutes prior to primary antibody incubation (overnight at 4°C). Either rabbit polyclonal serum directed against KdgM, or rabbit polyclonal serum directed against the CytC toxin, were used as primary antibodies. Pre-immune rabbit serum was used as a negative control. All antisera were diluted at 1∶1000 in PBS containing 0.1% bovine serum albumin. After the primary antiserum incubation, sections were washed with PBS containing 0.2% Tween. Primary antibodies were detected with fluorescent donkey anti rabbit IgG, labelled with Alexa fluor 488. This secondary antibody was applied for 1 hour at room temperature, diluted 1∶600 in BSA 0.1% in PBS. From this step onwards, all manipulations were carried out in the dark. Next, sections were washed with PBS-Tween, then rinsed with PBS and with several baths of tap water. The sections were left to dry on the bench, and then mounted using Gel Mount mounting medium, together with 4,6-diamidino-2-phenylindole (DAPI) for nuclear staining (3 µg per ml of medium). Sections were observed under an epifluorescence microscope (Olympus IX81), using specific emission filters: HQ535/50 for green signals (antibody staining), D470/40 for blue signals (DAPI) and HQ610/75 for red signals (unspecific tissue autofluorescence). Images were captured and processed using the Cell F Software (AnalySIS).

## Supporting Information

Figure S1 Panels A to F: Sections of aphids infected with wild type *D. dadantii* incubated with anti-KdgM antiserum (upper row) and with preimmune serum used as a negative control (lower row). Scale bar: 200 µm.
(JPG)

Figure S2 Panels A and B: Sections of aphids infected with wild type *D. dadantii* and incubated with anti-Cyt63 antiserum (upper row) and with preimmune serum used as a negative control (lower row). Panel C: Sections of Cyt-defective mutants (*Δcyt*), incubated with anti-Cyt63 antiserum (upper row) and preimmune serum used as a negative control. Scale bar: 200 µm.
(JPG)
