# Supplementary figures and images for: Dickeya dadantii, a Plant Pathogenic Bacterium Producing Cyt-Like Entomotoxins, Causes Septicemia in the Pea Aphid Acyrthosiphon pisum
Source: PLoS One. 2012 Jan 24;7(1):e30702. doi: 10.1371/journal.pone.0030702 (PMC3265518; doi:10.1371/journal.pone.0030702)

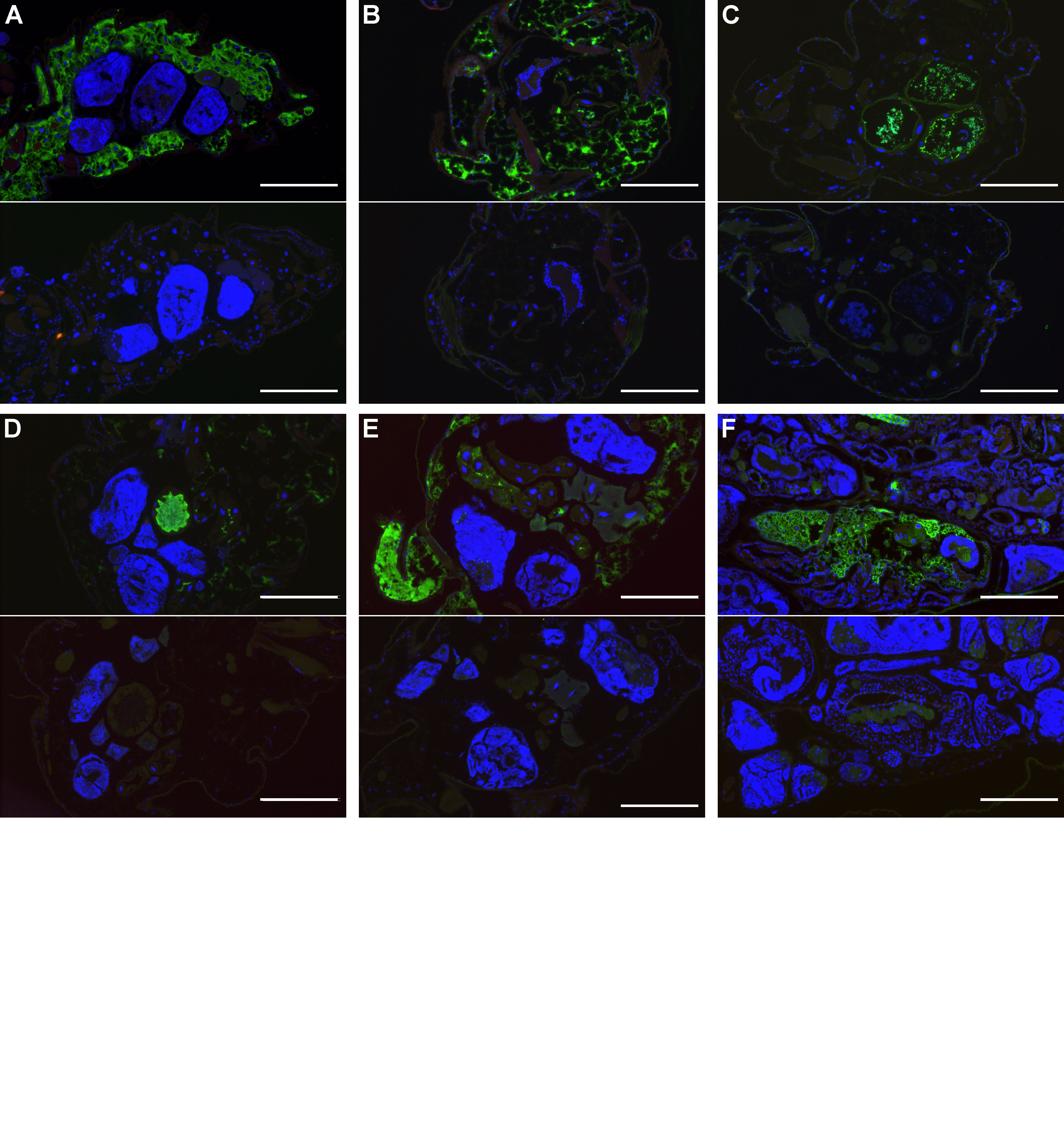

Supplement: Figure S1 — Panels A to F: Sections of aphids infected with wild type D. dadantii incubated with anti-KdgM antiserum (upper row) and with preimmune serum used as a negative control (lower row). Scale bar: 200 µm. (JPG) [file pone.0030702.s001.jpg]

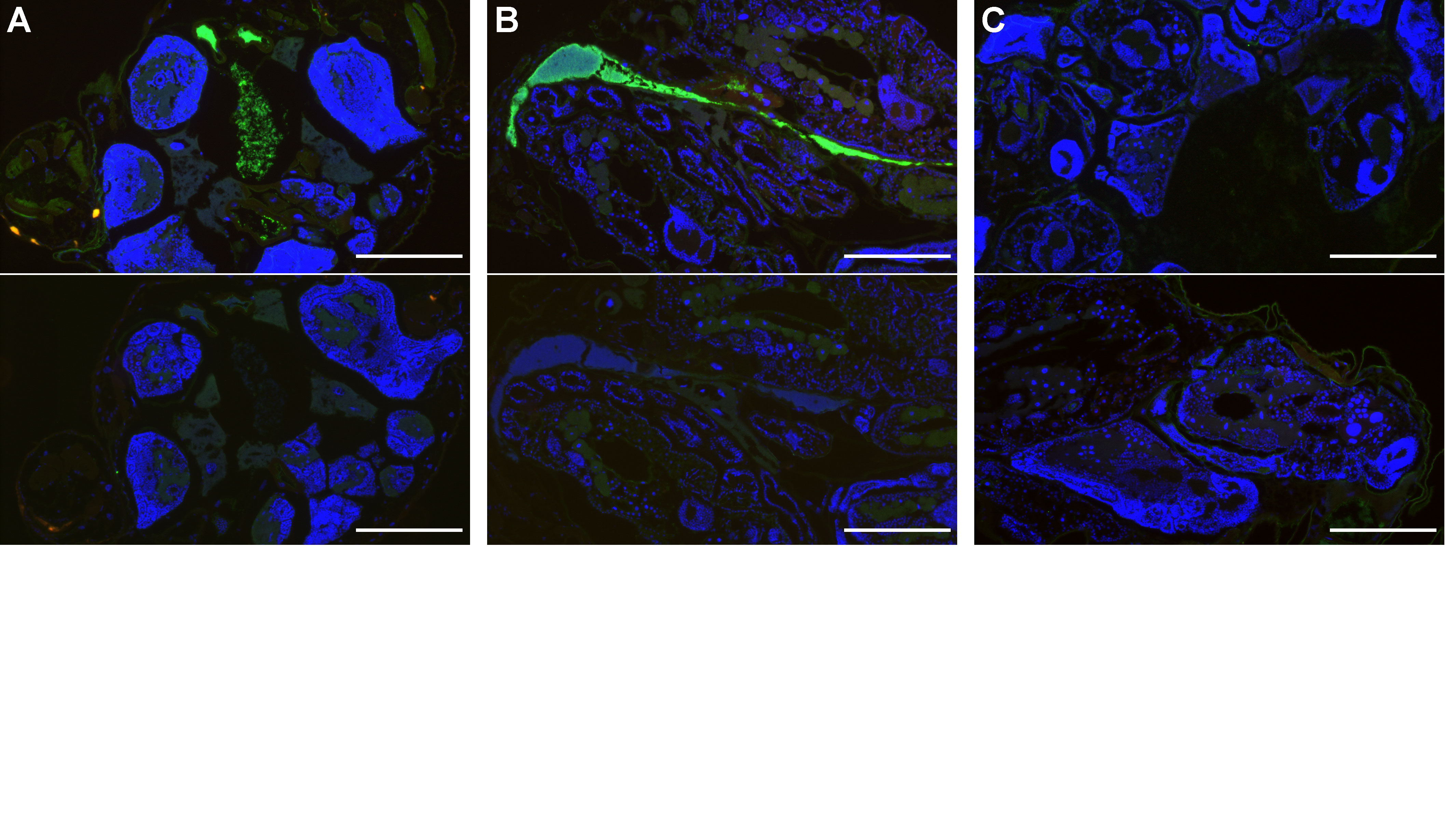

Supplement: Figure S2 — Panels A and B: Sections of aphids infected with wild type D. dadantii and incubated with anti-Cyt63 antiserum (upper row) and with preimmune serum used as a negative control (lower row). Panel C: Sections of Cyt-defective mutants (Δcyt), incubated with anti-Cyt63 antiserum (upper row) and preimmune serum used as a negative control. Scale bar: 200 µm. (JPG) [file pone.0030702.s002.jpg]
